# Long-term follow-up results of simultaneous integrated or late course accelerated boost with external beam radiotherapy to vaginal cuff for high risk cervical cancer patients after radical hysterectomy

**DOI:** 10.1186/s12885-015-1248-3

**Published:** 2015-04-11

**Authors:** Xin Wang, Yaqin Zhao, Yali Shen, Pei Shu, Zhiping Li, Sen Bai, Feng Xu

**Affiliations:** 1 Department of Abdominal Oncology, Cancer Center, West China Hospital, Sichuan University, Chengdu, Sichuan Province China; 2 State Key Laboratory of Biotherapy, West China Hospital, Sichuan University, Chengdu, Sichuan Province China; 3 Radiation and Physics Center, Cancer Center, West China Hospital, Sichuan University, Chengdu, Sichuan Province China

**Keywords:** Cervical cancer, Adjuvant chemoradiotherapy, Intensity modulated radiotherapy (IMRT), Volumetric modulated arc therapy (VMAT), Simultaneous integrated boost (SIB), Late course accelerated boost (LCAB)

## Abstract

**Background:**

To assess the safety and efficacy of simultaneous integrated boost (SIB) or late course accelerated boost (LCAB) with external beam radiotherapy (EBRT) to the vaginal cuff for high risk cervical cancer patients after radical hysterectomy.

**Methods:**

Between October 2009 and January 2012, patients with high risk cervical cancer who had undergone radical surgery followed by EBRT to the vaginal cuff were enrolled. Patients were treated with either intensity modulated radiotherapy (IMRT)/volumetric modulated arc therapy (VMAT) with SIB (arm A) or IMRT/VMAT to the pelvis followed by LCAB (arm B) to vaginal cuff. In arm A, the pelvic and boost doses were 50.4 Gy and 60.2 Gy in 28 fractions, respectively. In arm B, pelvic irradiation to 50 Gy in 25 fractions followed by a boost of 9 Gy in 3 fractions were delivered. Chemotherapy was given concurrently.

**Results:**

Overall, 80 patients were analyzed in this study (42 in arm A, 38 in arm B). In arm A and B, median follow-up was 37 and 32 months, respectively. The 3-year disease-free survival and overall survival in arms A vs B were 88.7% vs. 93.4% (p = 0.89), and 91.8% vs.100% (p = 0.21), respectively. The 3-year local-regional control and distant failure were 97.6% vs. 100% (p = 0.34), and 4.8% vs. 5.3% (p = 0.92), respectively. Grade 3–4 acute leukopenia and dermatitis were seen in 11 (26.2%) and 8 (19.0%) patients in Arm A, vs. 7 (17.8%) and 6 (15.8%) patients in Arm B, respectively (p > 0.05). Only Grade 1–2 chronic gastrointestinal (GI) and genitourinary (GU) toxicities were observed.

**Conclusions:**

Our results indicate that both SIB and LCAB to vaginal cuff for high risk cervical cancer patients after radical hysterectomy are associated with excellent survival, local control and low toxicity.

## Background

Cervical cancer constitutes the leading cause of cancer death among women in developing countries [1,2]. In early stage cervical cancer, surgery remains a major step of the therapeutic treatment. However, in women who are considered to be at high risk for recurrence due to additional risk factors, adjuvant radiotherapy following radical hysterectomy has been recommended [3-5].

Postoperative adjuvant radiotherapy for cervical cancer includes external beam radiation therapy (EBRT) and vaginal brachytherapy. Although there is no clear agreement as to the indications for performing vaginal brachytherapy after radical hysterectomy for cervical cancer, it is typically employed as a boost after EBRT [6]. The current National Comprehensive Cancer Network (NCCN) cervical cancer guidelines [7] and American Brachytherapy Society consensus guidelines both suggest that brachytherapy may be used as a boost to EBRT in postoperative patients with high risk factors, such as close or positive margins, a less than radical hysterectomy, large or deeply invasive tumors, extensive lymphovascular invasion, or parametrial or vaginal involvement [6]. However, in certain circumstances, vaginal brachytherapy may not be feasible due to patient refusal to undergo the procedure, unfavorable anatomy, coexisting medical conditions, or the lack of availability of brachytherapy in the institution. For these patients, EBRT can offer an alternative form of treatment. At the same time, with the rapid development of recent EBRT techniques, such as intensity-modulated radiotherapy (IMRT), volumetric-modulated arc therapy (VMAT), three dimensional- conformal radiotherapy (3D-CRT) andstereotactic radiotherapy, a radiation boost to the vaginal cuff and parametria can be achieved. Some studies explored these EBRT boost methods in patients with locally advanced cervical or endometrial cancer, and reported that delivering a total dose of 54–81.2 Gy was well tolerated and efficacious [8-12].

To patients after radical hysterectomy, the total EBRT boost dose prescribed to the vaginal cuff is lower than that employed in patients with unresected disease or gross residual tumor following a hysterectomy. As such, it may be reasonable and feasible to use EBRT to boost the vaginal cuff in high risk patients following a radical hysterectomy. This may be accomplished with a number of EBRT techniques, including IMRT, VMAT and 3D-CRT; it may also be delivered simultaneously or sequentially with whole-pelvic irradiation.

The purpose of this study is to report a single-institution experience using adjuvant EBRT to boost the vaginal cuff in high risk cervical cancer patients after radical hysterectomy, and compare two techniques for doing so, simultaneous integrated boost (SIB) with IMRT/VMAT and late course accelerated boost (LCAB) following pelvic IMRT/VMAT. To our knowledge, this is the first EBRT boost study in postoperative cervical cancer patients with high risk.

## Methods

### Patients

Patients treated at a single institution between October and January 2012 were evaluated if they underwent a radical hysterectomy with pelvic lymphadenectomy followed by adjuvant pelvic EBRT with EBRT vaginal cuff boost for a clinical stage IB-IIA cervical cancer, or for a stage IIB cervical cancer following neoadjuvant chemotherapy, but did not achieve a complete pathological response to neoadjuvant treatment. Patients were eligible for analysis if they had at least one of the following high risk factors after resection: close margins, large tumors (>4 cm), deep stromal invasion (defined as invasion into the deeper half of the cervical wall), extensive lymphovascular invasion, positive pelvic lymph nodes, or parametrial involvement. In addition, patients were required to have an Eastern Cooperative Oncology Group (ECOG) performance status of 0 or 1, a histologically negative surgical margin, and radiographically negative para-aortic lymph nodes. The EBRT boost to vaginal cuff was delivered as either IMRT/VMAT SIB (arm A) or IMRT/VMAT to the pelvis followed by LCAB with 3D-CRT (arm B) at the Department of Abdominal Oncology of West China Hospital of Sichuan University. The treatment protocols (arm A and arm B) were determined by the treating physicians. All patients were staged according to International Federation of Gynecology and Obstetrics (FIGO) protocol. The study was approved by the West China Hospital institutional review board. All patients provided written informed consent.

### Radiation therapy

All patients were immobilized in the supine position with abdominal body thermoplastic masks, and underwent helical computed tomography (CT, Siemens Sensation 4) at 3 mm slice thickness with intravenous contrast. All planning was performed using the Pinnacle treatment planning system (TPS). The clinical target volume (CTV) and organs at risk (OARs) (i.e., bladder, rectum, small bowel and femoral head) were contoured on sequential axial CT slices. CTV1 included the proximal two-thirds of the vagina, paravaginal soft tissue lateral to the vagina and pelvic lymph nodes (common, internal and external iliac, and presacral lymph node regions), and delineated according to the consensus guidelines for the delineation of the CTV in postoperative pelvic radiotherapy of endometrial and cervical cancer [13]. CTV2 included the proximal two-thirds of the vagina and paravaginal soft tissue lateral to the vagina. In order to decrease CTV geometric uncertainty, patients received instruction in bladder and rectum control. Patients were instructed to empty their bladder and then drink 500 ml of water one hour before simulation and each treatment, with the intention of having a moderately-full and comfortable bladder. Patients were also encouraged to move their bowels and to have an empty rectum in advance of their daily treatments. The planning target volumes (PTV1 and PTV2) were created by extending CTV1 and CTV2, respectively, using a margin of 10 mm in the axial plane except anterior to the rectum, where the margin was 5 mm. Extended treatment fields were not used. The rectum was contoured from the anus to the rectosigmoid flexure. The bladder was contoured as a \solid organ. In order to account for the displacement of the small bowel, the entire peritoneal cavity was contoured up to 1 cm above the superior extent of the PTV.

In arm A, 50.4 Gy/28 fractions and 60.2 Gy/28 fractions were delivered to PTV1 and PTV2, respectively, with an IMRT/VMAT SIB technique. In arm B, a dose of 50 Gy/25 fractions was delivered to PTV1 with an IMRT/VMAT technique, followed by a boost of 9 Gy/3 fractions delivered to PTV2 with 3D-CRT. All radiotherapy was delivered with 6 MV photons daily, 5 days per week. Inversely-planned step-and-shoot IMRT, VMAT and 3D-CRT plans generated. Cumulative dose-volume histograms were reviewed. Plans were acceptable if the prescribed dose covered >95% of the PTV and no more than 1 cc received >107% of the prescribed dose. Typical normal tissue constraints were as follows: <50% bladder was to receive 50 Gy, <50% rectum was to receive 50 Gy, <40% of small bowel was to receive 40 Gy, and <5% of the femoral heads were to receive 50 Gy.

Adjuvant radiotherapy began within 3 months after surgery. All patients received 4 cycles of adjuvant chemotherapy concurrently with their radiotherapy, using either paclitaxel & cisplatin (TP), 5-FU & cisplatin (FP) or bleomycin & cisplatin (BP). Patients with stage IIB disease had neoadjuvant chemotherapy to down-stage the tumor.

### Follow-up

Adverse events (AEs) were assessed on a weekly basis during treatment using the National Cancer Institute Common Terminology Criteria for Adverse Events, version 3.0 (CTCAE v 3.0). After treatment, patients were followed up every 3 months for 2 years, then every 6 months for the following 3 years. Follow-up assessments were based on either physical examination by the radiation oncologists or CT scans.

### Statistics

We estimated local-regional control (LC), distant failure (DF), and AEs using cumulative incidence functions. Disease-free survival (DFS) and overall survival (OS) were estimated using the Kaplan-Meier method; comparisons between groups were made using the log-rank test. DFS was defined as the time between hysterectomy and first evidence of disease recurrence or the most recent follow-up. OS was defined as the time between hysterectomy and death from any cause or the most recent follow-up. For the purposes of DFS, patients were censored at the time of last follow-up or death without any progression of disease. For the purposes of OS, patients were censored at the time of last follow-up. Differences between the two arms were evaluated using a two-sample t-test for continuous variables and Pearson’s chi-square test was used for categorical data. Statistical analysis was conducted using PASW Statistics (SPSS, IBM Corporation). For all analyses, a *P* value of <0.05 was considered statistically significant. All tests of statistical significance were 2-sided.

## Results

### Patients

Overall, a total of 80 patients were analyzed in this study (42 in arm A, 38 in arm B). Patient characteristic data are summarized in Table 1. The median follow- up interval was 37 months (range, 15–49) in arm A and 32 months (range, 16–47) in arm B. The median age was 45 (range, 33–57 years) and 44 (range, 33–69) years in arms A and B, respectively. There were no significant differences between the baseline patient characteristics of the two arms (p > 0.05) (Table 1).

**Table 1:** **Baseline patient characteristics**

Characteristic	Arm A (n = 42)	Arm B (n = 38)	p value
	N (%)	N (%)	
Age (years)			0.69
Range	33-57	33-69	
Median	45	44	
	n (%)	n (%)	
FIGO stage			0.71
IB	12 (28.6)	9 (23.7)	
IIA	15 (35.7)	12 (31.6)	
IIB	15 (35.7)	17 (44.7)	
Histology			0.40
Squamous	40 (95.2)	38 (100)	
Adenocarcinoma	1 (2.4)		
Neuroendocrine	1 (2.4)		
Histologic grade			0.64
G1: Well differentiated	3 (7.1)	1 (2.6)	
G2: Moderately differentiated	7 (16.7)	6 (15.6)	
G3: Poorly differentiated	32 (76.2)	31 (81.6)	
Lymph node metastases			0.59
+	12 (28.6)	13 (34.2)	
-	30 (71.4)	25 (65.8)	
CLS			0.89
+	15 (35.7)	13 (34.2)	
-	27 (64.3)	25 (65.8)	
Stromal invasion			0.15
Superficial half	6 (14.3)	3 (7.8)	
Deep half	22 (52.4)	28 (73.7)	
Whole stroma	14 (33.3)	7 (18.4)	

CLS: capillary lymphatic space.

The treatment characteristics are summarized in Table 2. There were 11 and 12 patients treated with VMAT, as well as 31 and 26 patients treated with IMRT in arms A and B, respectively. Image-guided radiotherapy was used in 8 and 12 cases in arms A and B, respectively (Table 2). 36 patients in arm A and 34 patients in arm B were also treated with chemotherapy. 16 and 19 patients with stage II in arm A and B underwent neoadjuvant chemotherapy, respectively (Table 2). All of these patients achieved tumor shrinkage and then received radical hysterectomy with pelvic lymphadenectomy.

**Table 2:** **Treatment characteristics**

Treatment	Arm A (n = 42)	Arm B (n = 38)	p value
	N (%)	N (%)	
Radiotherapy			0.60
VMAT	11 (26.2)	12 (31.6)	
IMRT	31 (73.8)	26 (68.4)	
IGRT	8 (19.0)	12 (31.6)	0.19
Chemotherapy Regimen			0.78
TP	15 (35.7)	14 (36.8)	
BP	13 (31.0)	15 (39.5)	
FP	8 (19.0)	5 (13.2)	
No Chemotherapy	6 (14.3)	4 (10.5)	
Neoadjuvant Chemotherapy	16 (38.1)	19 (50.0)	0.28

VMAT: volumetric modulated arc therapy; IMRT: intensity modulated radiotherapy; IGRT: Image-guided radiation therapy; TP: paclitaxel & cisplatin; BP: bleomycin & cisplatin; FP: 5-FU & cisplatin.

The biological equivalent dose (BED) to the vaginal cuff was calculated with the linear-quadratic model to be 73.14 Gy in arm A and 71.7 Gy in arm B, assuming a 2 Gy/fraction schedule, with α/β = 10. Concurrent chemoradiotherapy was well tolerated, with only 4 (9.5%) and 3 (7.9%) of treatment interruptions in arms A and B, respectively.

### Outcomes

**Figure 1:**
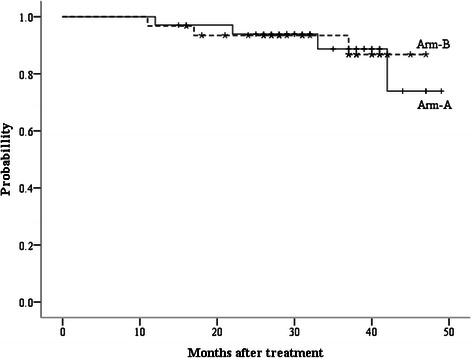
Disease-free survival curves for arm A (IMRT/VMAT SIB) and B (IMRT/VMAT followed by LCAB).

**Figure 2:**
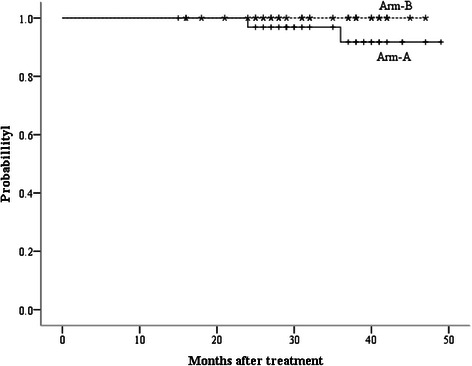
Overall survival curves for arm A (IMRT/VMAT SIB) and B (IMRT/VMAT followed by LCAB).

In this study, local failure alone occurred in 1 patient in arm A, who had an isolated vaginal cuff recurrence, while there was no local-regional recurrence observed in arm B. The 3-year LC rates were 97.6% for arm A and 100% for arm B (p = 0.34). Distant metastasis occurred in 2 patients in each arm. In arm A, the sites of distant metastasis were retroperitoneal nodes and supraclavicular nodes, while in arm B, the lung and liver were involved. The 3-year DF were 4.8% for arm A and 5.3% for arm B (p = 0.92). Figure 1 shows the DFS of two arms. The 1, 2, 3-year DFS for arms A and B were 97.1% vs. 96.8%, 93.9% vs. 93.4%, and 88.7% vs. 93.4%, respectively. There was no significant difference between two groups (p = 0.89). During follow-up, there was only 1 patient death, in arm A. The 3-year OS for arm A and B were 91.8% and 100%, respectively (p = 0.21) (Figure 2).

### Adverse events

Acute treatment-related Grade 3–4 AEs during treatment were shown in Table 3. Leukopenia was the most common Grade 3–4 acute AEs, and was seen in 11 (26.2%) and 7 (17.8%) patients in Arm A and B, respectively (Table 2). Grade 3 dermatitis was seen in 8 (19.0%) and 6 (15.8%) patients in two arms, respectively, and it was the second common AEs in this study (Table 3). No Grade 4 acute dermatitis was seen. The differences in AEs between the two arms were not significant (p > 0.05) (Table 3). Late AEs were very mild in both arms (Table 4). Only Grade 1–2 chronic gastrointestinal (GI) and genitourinary (GU) toxicities were observed in this study. Grade 2 chronic GI toxicity was seen in 2 patients in arm A and 1 in arm B, while Grade 2 chronic GU toxicity was only seen in 1 patient in arm A (Table 4). All patients were successfully managed conservatively or symptomatically, and were symptom-free at last follow-up.

**Table 3:** **Acute grade 3–4 adverse events (AEs) in arms A and B occurring during concurrent chemoradiotherapy**

AEs	Arm A (n = 42)	Arm B (n = 38)	p value
	N (%)	N (%)	
Grade 3			
Leukopenia	9 (21.4)	6 (15.8)	0.42
Neutropenia	5 (11.9)	2 (5.3)	0.29
Thrombocytopenia	1 (2.4)	0 (0)	0.34
GI	4 (9.5)	1 (2.6)	0.20
Dermatitis	8 (19.0)	6 (15.8)	0.70
Grade 4			
Leukopenia	2 (4.8)	1 (2.6)	0.62
Neutropenia	1 (2.4)	0 (0)	0.34

GI: gastrointestinal toxicity.

**Table 4:** **Chronic AEs observed in arms A and B**

AEs	Arm A (n = 42)	Arm B (n = 38)	p value
	N (%)	N (%)	
Grade 1			
GI	2 (4.8)	1 (2.6)	0.62
GU	1 (2.4)	2 (5.3)	0.50
Grade 2			
GI	2 (4.8)	1 (2.6)	0.62
GU	1 (2.4)	0 (0)	0.34
Grade 3	0 (0)	0 (0)	
Grade 4	0 (0)	0 (0)	

GI: gastrointestinal toxicity; GU: genitourinary toxicity.

## Discussion

It was previously reported that based on the Surveillance, Epidemiology, and End Results (SEER) database, the rate of brachytherapy use for cervical cancer in the United States fell from 83% in 1988 to 43% in 2003, and one of the most important reasons was increased utilization of highly conformal radiation therapy techniques such as IMRT [14]. The recommended dose to the vaginal cuff for postoperative high risk cervical cancer patients is 12 Gy in 2 fractions of high dose rate (HDR) brachytherapy following 50.4 Gy of EBRT. This is much lower than the dose recommended for unresected cervical cancer patients [6]. Accordingly, it’s feasible to facilitate the adoption of EBRT boost to the vaginal cuff as an alternative to brachytherapy for postoperative cervical cancer. And it is also recommended that an additional 10-15Gy highly conformal EBRT boost to the vaginal cuff may be considered to replace brachytherapy following whole-pelvic EBRT [15]. IMRT has been frequently used for cervical cancer in recent years, and has been demonstrated to be able to provide a relatively precise dose distribution to the CTV while reducing the dose to OARs, consequently decreasing complications with possible enhancement or no loss of curative effect in postoperative cervical cancer patients [16-21]. VMAT is another effective highly precise radiotherapy technique available in recent years. Many studies had reported the encouraging results of this technique in several kinds of cancers [22-25]. EBRT boost techniques explored in this study were IMRT/VMAT SIB and LCAB with 3D-CRT following pelvic IMRT/VMAT. Both techniques can perform the boost to the vaginal cuff. To our knowledge, this is the first study to report the safety and efficacy of an EBRT boost to the vaginal cuff, and make a comparison between two boost techniques in postoperative cervical cancer patients with high risk factors.

In this study, the 3-year DFS and OS for the SIB group were 88.7% and 91.8%, respectively, which were not significantly different from those in LCAB group (93.4%, and 100%), with p = 0.89 and p = 0.21, respectively. Local failure was only observed in 1 patient in the SIB group, and was isolated to the vaginal cuff. Our results show that both the SIB and LCAB techniques can provide excellent local-regional control, DFS and OS. These results also compare well with others reported in the literature. Some previous studies delivered adjuvant radiotherapy with a conventional radiotherapy technique and without a brachytherapy boost, and reported local-regional recurrence rates and 4–5 year OS of 8.6-21.6% and 71–96.7%, respectively [3,26-28]. Other studies performed adjuvant IMRT without a vaginal cuff boost [29], and reported 3- and 5-year DFS and OS of 91.2% and 91.1%, respectively [29]. Our results compare well with studies that performed adjuvant pelvic radiotherapy with a vaginal brachytherapy boost [30-32]. Chen et al. performed adjuvant IMRT (50.4 Gy in 28 fractions) followed by brachytherapy (6 Gy in 3 insertions); and reported a 3-year local-regional control, DFS and OS of 93%, 78% and 98%, respectively [30]. Pieterse et al. delivered conventional four-field radiotherapy and brachytherapy to post-operative, high risk cervical cancer patients [32]. The 5-year cancer-specific survival and DFS in that study were 86% and 85%.

The extent of hematologic toxicity can be affected by chemotherapy regimen as well as radiotherapy. When adjuvant conventional radiotherapy and concurrent chemotherapy were performed, Grade 3–4 leukopenia in 43 (35.2%), granulocytopenia in 35 (28.7%), and thrombocytopenia in 1 (0.8%) patients were reported [3]. Several studies demonstrated that hematologic toxicity could be reduced with IMRT in comparison to conventional radiotherapy [19,30,31,33,34]. Chen et al. compared the toxicity of adjuvant IMRT and conventional radiotherapy followed by brachytherapy with concurrent weekly cisplatin [31]. This study demonstrated that Grade 2 hematologic toxicity in the IMRT and conventional radiotherapy groups were observed in 9 (27%) and 11 (31%) patients, while Grade 3 hematologic toxicity were noted in 2 (6%) and 3 (9%) patients, respectively. Mell et al. treated cervical cancer patients with IMRT and concurrent cisplatin, and observed Grade 3–4 anemia, granulocytopenia and leukopenia in 3 (8.1%), 1 (2.7%), and 4 (10.8%) patients, respectively [35]. There were more Grade 3–4 hematologic toxicities reported in our study. Leukopenia was the most common Grade 3–4 acute AE in our study, and was observed in 11 (26.2%) and 7 (17.8%) patients in arms A and B, respectively (Table 2). There were no significant differences between the two arms. The adjuvant concurrent chemotherapy used in our study was 4 cycles of TP, BP or FP, which may cause more hematologic toxicity than weekly cisplatin alone. Similar results were reported by another study, and Grade 3–4 hematological toxicity was 32.3% when concurrent adjuvant FP chemotherapy was administered with IMRT without vaginal cuff boost [29].

As to the GI and GU toxicities, Chen et al. reported that IMRT had significant lower acute Grade 1–2 GI (36% vs. 80%, *p* = 0.00012), and GU (30% vs. 60%, *p* = 0.022) toxicities when compared with the conventional radiation group [27]. Furthermore, they demonstrated that the IMRT group also resulted in lower rates of chronic Grade 1–3 GI (6 vs. 34%, *p* = 0.002), and GU (9 vs. 23%, *p* = 0.231) toxicities [31]. Similar results were also reported by other studies [19,30,33,34]. In our study, we demonstrated that concurrent chemotherapy with the SIB and LCAB techniques was well tolerated with low incidences of acute and chronic GI and GU toxicity (Tables 3 and 4). Our results were similar to other studies where no boost was performed after pelvic IMRT. In one such study, Folkert et al. reported that 2.9% acute Grade 3 GI toxicity, and no acute Grade 3 or higher GU toxicity was observed, and that chronic Grade 1 GI and GU toxicity occurred in 5 (14.7%) and 4(11.8%) patients, while chronic Grade 2 GU toxicity occurred in 1(2.9%) patient [29].

The weaknesses of this study are due to its retrospective and single-institution nature, the small sample size, and the lack of standardization in the chemotherapy. Moreover, the difference in the efficacy of an EBRT versus a brachytherapy boost to the vaginal cuff cannot be compared directly. However, to our knowledge, this is the first study to report the safety and efficacy of an EBRT boost to the vaginal cuff, and make comparison between two boost techniques in postoperative high-risk cervical cancer patients.

## Conclusions

In conclusion, the current study suggests that good oncologic outcomes are achievable with both IMRT/VMAT SIB and IMRT/VMAT followed by LCAB to the vaginal cuff and concurrent chemotherapy for postoperative high risk cervical cancer patients. Both techniques are safe and feasible, with good local tumor control, good DFS and OS, and well tolerated. There were no significant differences between the two the radiation techniques.
